# Discriminant models for the prediction of postponed viral shedding time and disease progression in COVID-19

**DOI:** 10.1186/s12879-022-07338-x

**Published:** 2022-04-11

**Authors:** Wen-Yang Li, Daqing Wang, Yuhao Guo, Hong Huang, Hongwen Zhao, Jian Kang, Wei Wang

**Affiliations:** 1 Respiratory and Critical Care Department, The First Hospital of China Medical University, Shenyang, China; 2 Department of Respiratory Disease, Liaoning Province Peoples’ Hospital, Shenyang, China; 3 Department of Mathematics and Statistics, Xian Jiaotong University, Xian, 710049 China

**Keywords:** Prognostic discriminant model, Postponed viral shedding time, Disease progression, COVID-19

## Abstract

**Background:**

COVID-19 infection can cause life-threatening respiratory disease. This study aimed to fully characterize the clinical features associated with postponed viral shedding time and disease progression, then develop and validate two prognostic discriminant models.

**Methods:**

This study included 125 hospitalized patients with COVID-19, for whom 44 parameters were recorded, including age, gender, underlying comorbidities, epidemiological features, laboratory indexes, imaging characteristics and therapeutic regimen, et al. Fisher's exact test and Mann–Whitney test were used for feature selection. All models were developed with fourfold cross-validation, and the final performances of each model were compared by the Area Under Receiving Operating Curve (AUROC). After optimizing the parameters via L_2_ regularization, prognostic discriminant models were built to predict postponed viral shedding time and disease progression of COVID-19 infection. The test set was then used to detect the predictive values via assessing models’ sensitivity and specificity.

**Results:**

Sixty-nine patients had a postponed viral shedding time (> 14 days), and 28 of 125 patients progressed into severe cases. Six and eleven demographic, clinical features and therapeutic regimen were significantly associated with postponed viral shedding time and disease progressing, respectively (*p* < 0.05). The optimal discriminant models are: y_1_ (postponed viral shedding time) = − 0.244 + 0.2829x_1_ (the interval from the onset of symptoms to antiviral treatment) + 0.2306x_4_ (age) + 0.234x_28_ (Urea) − 0.2847x_34_ (Dual-antiviral therapy) + 0.3084x_38_ (Treatment with antibiotics) + 0.3025x_21_ (Treatment with Methylprednisolone); y_2_ (disease progression) = − 0.348–0.099x_2_ (interval from Jan 1st,2020 to individualized onset of symptoms) + 0.0945x_4_ (age) + 0.1176x_5_ (imaging characteristics) + 0.0398x_8_ (short-term exposure to Wuhan) − 0.1646x_19_ (lymphocyte counts) + 0.0914x_20_ (Neutrophil counts) + 0.1254x_21_ (Neutrphil/lymphocyte ratio) + 0.1397x_22_ (C-Reactive Protein) + 0.0814x_23_ (Procalcitonin) + 0.1294x_24_ (Lactic dehydrogenase) + 0.1099x_29_ (Creatine kinase).The output ≥ 0 predicted postponed viral shedding time or disease progressing to severe/critical state. These two models yielded the maximum AUROC and faired best in terms of prognostic performance (sensitivity of78.6%, 75%, and specificity of 66.7%, 88.9% for prediction of postponed viral shedding time and disease severity, respectively).

**Conclusion:**

The two discriminant models could effectively predict the postponed viral shedding time and disease severity and could be used as early-warning tools for COVID-19.

**Supplementary Information:**

The online version contains supplementary material available at 10.1186/s12879-022-07338-x.

## Background

**Summary:** this study fully characterizes the clinical features associated with postponed viral shedding time and disease progression, then develop and validate two prognostic models with satisfactory discriminant performance.

The prevalence of coronavirus disease 2019 (COVID-19) has put a huge burden to medical resources [1]. Although patients with COVID-19 infection mostly manifested as non-severe cases, it can also cause life-threatening conditions before or during hospitalization, such as severe pneumonia, adult respiratory distress syndrome or multiple organ failure, which are all related to worse outcomes [2]. Compare to the other epidemiological disease, such as the previous outbreaks of SARS-CoV and MERS-CoV, COVID-19 progresses and spreads more rapidly, with peculiar epidemiological traits. High viral loads of SARS-CoV-2 were observed in the upper respiratory specimens of patients with little or no symptoms, this indicated that inapparent-transmission plays a major but underestimated role in sustaining the outbreak of COVID-19 [3].

Since the first case emerged in Liaoning province in Jan 22th, 2020, there is an urgent need to construct a simple, efficient and accurate “early-warning prediction model” for disease progression at early stage once the patients were admitted to the hospital. This will facilitate the medical staff to make critical time-sensitive decisions regarding patients and treatments. Traditional evaluation scoring tools, such as CURB-65, qSOFA, and NEWS, could be adopted to assess disease severity, but not for the early assessment of COVID-19 severity [4]. Thus, to promptly predict and identify patients with postponed viral shedding time and disease severity is required but challenging. To date, dozens of prediction models of COVID-19 have been established to respond quickly to this healthcare crisis [5, 6]. Unfortunately, the quality of some of the identified models is uniformly poor and none can be recommended for clinical use, as demonstrated by one systemic review [7]. This study aimed to fully characterize the demographic, epidemiological, clinical features and therapeutic regimens and to detect their association with postponed viral shedding time and disease progression among patients with COVID-19 in Liaoning province, China. Furthermore, another purpose of this study was to specifically design and validate two prognostic discriminant models incorporating the associated features. These new mathematical models can serve as early-warning prediction tools to estimate the postponed viral shedding time and to identify the risk of progressing to severe stage in advance among patients with COVID-19 infection.

## Methods

### Study design

This retrospective multi-center cohort study included consecutive patients, they were laboratory-confirmed with COVID-19 infection and enrolled from Jan 22th to Mar 22th 2020 in eight designated hospitals throughout Liaoning province. All the enrolled patients were diagnosed with COVID-19 according to the WHO interim guidance [8]. Laboratory confirmation of COVID-19 was achieved by the nucleic acid test using real-time reverse transcriptase-polymerase chain reaction (RT-PCR) assay at Liaoning municipal Center for Disease Prevention and Control (CDC). Samples were collected using a nose swab and/or throat swab from each suspected patient. This study was approved by the human research ethics committee of the first hospital of CMU (committee’s reference number: AF-OG-20-1.1-02), Shenjing hospital of CMU (committee’s reference number: 2020041002), Liaoning province peoples’ hospital, Shenyang Sixth People's Hospital, Jinzhou Infectious Disease Hospital, Tieling infectious Disease Hospital, Fuxin Infectious Disease Hospital, Central Hospital of Huludao City. The other committee’s reference number were not available. All the written informed consent was waived. Permissions were obtained to access the data, as this was included as a part of the formal ethics approval. The data used in this study was anonymized before its use.

### Data collection

The date of disease onset (defined as the day when any symptom was noticed by the patients) and hospital admission date, the first day for nucleic acid detected to be positive or negative were all recorded. All the patients were hospitalized, and the clinical outcomes were monitored for at least 8 weeks. All the clinical data on epidemiology (recent exposure history), symptoms, signs, underlying comorbidities, laboratory results (on admission), imaging findings (on admission) and clinical progression were recorded and retrospectively double-extracted from electronic medical records, with two independent reviewers extracted the data and evaluated the eligibility of the original data. Long-term exposure to Wuhan was defined by Wuhan residence or study, work for at least one-month in Wuhan. Short-term exposure to Wuhan was defined by meeting, transfer or travel history to Wuhan temporarily. Considering that one of the isolation release and discharge criteria for hospitalized patients is a sputum/oral swab testing negative twice with 24 h interval [5], virus detection was repeated twice every 24 h. It was deemed as viral clearance when virus was detected to be negative for two consecutive times.

Regarding antiviral treatment, 14 patients with confirmed COVID-19 were treated with lopinavir–ritonavir (400 mg/100 mg), 11 with arbidol (200 mg t.i.d.). Dual-antiviral therapy of nebulized Interferon-α (IFN-α) (5 mU b.i.d.) with lopinavir–ritonavir or arbidol were used in 42 and 8 patients. Triple-antiviral therapy of IFN-α, lopinavir–ritonavir and arbidol were used in 20 patients. Other therapies such as antibiotics and corticosteroids were used in 38 and 22 patients.

### Definition of disease progression and postponed virus shedding time

Disease progression were recorded for at least 8 weeks after admission. Severity of COVID-19 was defined according to the American Thoracic Society (ATS) guidelines for community-acquired pneumonia (CAP) [9]. Severe/critical cases of COVID-19 should meet one major criterion (septic shock with need for vasopressors or respiratory failure requiring mechanical ventilation) or at least three minor criteria: (a) respiratory distress with respiratory frequency ≥ 30/min; (b) oxygenation index (partial pressure of oxygen/inspired oxygen fraction, PaO_2_/FiO_2_) ≤ 250 mmHg; (c) multilobe infiltrates, confusion/disorientation; (d) uremia (blood urea nitrogen ≥ 20 mg/dL); (e) leukopenia (white blood cell count < 400 cells/μL); (f) thrombocytopenia (platelet count < 100,000/μL); (g) hypothermia (body temperature < 36 ºC);(h) hypotension requiring aggressive fluid resuscitation. Non-severe patients were defined as a confirmed case with fever, respiratory symptoms, with or without radiographic evidence of pneumonia.

The median viral shedding time was 14 days (IQR, 11–19), those ≥ 14 days were deemed as postponed virus shedding.

### Variables

Discriminant variables for disease severity and postponed viral shedding time were determined according to the assessment of the existing medical records (Additional file 1: Table S1) listed as follows: the interval from onset of symptoms to antiviral treatment (x_1_),interval from Jan 1st,2020 (the day for the first case emerged in Liaoning) to individualized onset of symptoms (x_2_), gender (x_3_), age (x_4_), imaging characteristics (x_5_);long-term exposure to Wuhan (x_6_), local transmitted history (x_7_), short-term exposure to Wuhan (x_8_); respiratory symptoms (x_9_), digestive symptoms (x_10_), general malaise (x_11_), fever (x_12_); comorbidities with chronic respiratory disease (x_13_), hypertension (x_14_), diabetes mellitus (x_15_), surgery history (x_16_), other comorbidities (x_17_), White blood cell (WBC) (x_18_), lymphocyte counts (x_19_), neutrophil counts (x_20_), neutrophil/lymphocyte ratio (N/L) (x_21_), C-Reactive Protein (CRP) (x_22_), Procalcitonin (PCT) (x_23_), Lactic dehydrogenase (LDH) (x_24_), Aspartate amino transferase (AST) (x_25_), Alanine aminotransferase (ALT) (x_26_), Creatinine (Cr) (x_27_), Urea nitrogen (Urea) (x_28_), Creatine kinase (CK) (x_29_), oxygenation index(x_30_),disease severity (x_31_), treatment with Lobinavi/ritonavir alone (x_32_),treatment with Arbidol alone (x_33_), combined treatment of nebulized IFN-α with lopinavir–ritonavir (x_34_), combined treatment of nebulized IFN-α, with Arbidol (x_35_), combined treatment of nebulized IFN-α, lopinavir–ritonavir and Arbidol (x_36_), treatment with Oseltamivir phosphate alone (x_37_), Treatment with antibiotics (x_38_), treatment with ribavirin (x_39_), treatment with Chinese traditional medicine (Xuebijing) (x_40_), treatment with Methylprednisolone (x_41_), treatment with γ-globulin (x_42_),antiviral treatment course (x_43_). The outcomes were postponed viral shedding time (y_1_) and disease progression (y_2_).

Discriminate factors were also quantitatively assigned, some variables such as the imaging characteristics were assigned from 0, and the order was based on their influence on the progression of disease.

### Establish the optimal discriminant models for disease progression and postponed viral shedding time

Setting disease progression and postponed viral shedding time as the goal for discriminant models, logistic regression, linear discriminant analysis, K-nearest neighbor, support vector machine (SVM) and decision tree were constructed through Python 3.6 software (Numpy and Sklearn package). The dataset was split 4:1 by stratified random sampling and four-fifths was used as a training group to establish models. After comparing effectiveness among the models by analyzing total accuracy in both training and testing data set, the most optimal discriminant models incorporating multiple related factors were established to reflect the probability of disease progressing to severe stage or postponed viral shedding time. The models were constructed using the output as an outcome, while the output ≥ 0 indicated disease progressing to severe/critical stage or postponed viral shedding time.

### Verification of the discriminant models

The precision of the prediction models was further evaluated and validated. Due to the resampling methods, bootstrapping or cross-validation were more powerful than splitting the sample for internal validation. We applied cross-validation on the basis of random stratification. By comparing the area under the receiver-operating characteristic curve (AUROC) value, sensitivity, specificity, accuracy and recall rate, et al., the optimal primary screening model was chosen. Multicollinearity was calculated to assess the feasibility of the optimal model. Receiver operating characteristic (ROC) curves and confusion matrix were constructed to describe the screening effectiveness of the optimal model. By analyzing total accuracy, the optimal discriminant model was chosen.

### Statistical analysis

Categorical variables were summarized as frequencies and percentages. Continuous variables were described using median and interquartile ranges (IQR) values. Data were then compared between groups divided by disease severity (non-severe vs. severe group) or viral shedding time (with cutoff value 14 days) using F test for continuous variable, or by Mann–Whitney test or χ^2^ test for categorical data. Features significantly different (*p* < 0.05) in both algorithms were selected into the models. In order to eliminate the overfitting effect and regularize the models, z-score standardization [x^*^ = (x − mean)/standard deviation] was conducted on all continuous variables in the data set. Thus, each corresponding feature was converted into a normal distribution with mean value of 0 and variance of 1, to eliminate the dimensional influence.

## Results

### Demographic and clinical characteristics of patients with COVID-19 infection

A total of 125 hospitalized patients diagnosed as COVID-19 infection were included in this study. Disease progression was recorded during the 8-weeks follow-up after admission. Ten patients were categorized into mild cases, 97 developed into moderate cases and 28 patients developed into severe cases (including 3 critically ill) during hospitalization. Symptoms were shown in Table 1. Among 125 patients enrolled, 75 (62%) were imported cases (with an exposure history to Wuhan), which further divided into 37 (29.6%) short-term exposure (meeting, transfer or travel et al.) and 38 (30.4%) long-term exposure (residence or study, work in Wuhan) history to Wuhan. The rest 50 (40%) cases had no history of Wuhan exposure, thus were categorized into local transmitted cases who had contact with symptomatic cases. The median age for all patients were 44 years (IQR, 34–57), for non-severe patients was 41 years old (IQR, 34–55), and for severe patients was 50 years old (IQR, 38–63). About half (55.2%) of patients were male. Among all patients, up to 61 patients had at least one underlying comorbidities, the most common of which were chronic diseases, such as hypertension, et al. The median interval from the onset of symptoms to hospital admission was 4 days (IQR, 2–7). The median viral shedding time was 14 days (IQR, 11–19).

**Table 1:** Demographic and baseline characteristics of patients with different severities and virus shedding of COVID-19 infection

	Total (N = 125)	Non-severe (N = 97)	Severe/critical (N = 28)	*p*	Virus shedding < 14 days (N = 56)	Virus shedding ≥ 14 days (N = 69)	*p*
	Median (range)				Median (range)		
**Age**	44 (34–57)	41 (34–55)	50 (38–63)	0.001	41 (29–54)	45 (37–59)	0.07

Gender	No. (%)			*p*	No. (%)		*p*
Male	69 (55.4%)	51 (52.6%)	18 (64.3%)	0.49	34 (59.6%)	35 (50.7%)	0.50
Female	56 (44.6%)	46 (47.4%)	10 (35.7%)		22 (40.4%)	34 (49.3%)	

Epidemic features	No. (%)				*p*	No. (%)		*p*
Long-term exposure to Wuhan	38 (30.4%)	33 (34.0%)		5 (17.9%)	0.16	16 (29.8%)	22 (31.9%)	0.84
Short-term exposure to Wuhan	37 (29.6%)	22 (22.7%)		15(53.6%)	0.04	19 (33.3%)	18 (26.1%)	0.45
Local transmitted cases	50 (40%)	42 (43.3%)		8 (28.6%)	0.65	21 (36.8%)	29 (42.0%)	0.64
**Comorbidity-No. (%)**	61 (37.6%)	31 (32.0%)		28 (100%)	0.000	22 (38.6%)	39 (56.5%)	0.05
Chronic airway disease	5 (4.1%)	3 (3.1%)		2 (7.1%)	0.18	2(3.5%)	3 (4.3%)	0.12
Hypertension	22 (12.0%)	15 (15.5%)		7 (25%)	0.14	8 (15.8%)	14 (20.3%)	0.40
Diabetes mellitus	9 (4.0%)	6 (6.2%)		3 (10.7%)	0.88	2 (3.5%)	7 (10.1%)	0.07
Surgery history	12 (10.4%)	7 (7.2%)		5 (17.9%)	0.15	5 (8.8%)	7 (10.1%)	0.80
Others (ex. kidney stone)	13 (2.4%)	8 (8.2%)		5 (17.9%)	0.24	5 (8.8%)	8 (11.6%)	0.62
**Interval from symptom onset to diagnosis (days)**	4 (2–7)	4 (2–7)		3 (1.7–7.9)	0.01	5 (1–9)	3 (2–6)	0.07
**COVID-19 viral RNA shedding time (days)**	14 (11–19.3)	14 (11–18)		16 (13–22)	0.15	10 (8–12)	19 (15–25)	< 0.001
**Interval between outbreak in Liaoning and diagnosis (days)**	11 (7–16)	13 (7.5–17.5)		10 (5.5–14.0)	0.01	12 (7–18)	10 (7–16)	0.28

Symptoms	No. (%)			*p*	No. (%)		*p*
Fever	103 (82.4%)	75 (77.3%)	28 (100%)	0.006	44 (77.2%)	59 (85.5%)	0.61
Cough	78 (62.4%)	54 (55.7%)	24 (85.7%)	0.004	38 (66.7%)	40 (58.0%)	0.54
Dyspnea/chest tightness	31 (%)	5 (5.2%)	26 (92.9%)	< 0.001	19 (33.3%)	22 (31.9%)	0.87
Diarrhea/nausea	15 (12%)	12 (12.3%)	3 (10.7%)	0.36	7 (12.3%)	8 (11.6%)	0.91
Fatigue	35 (28%)	22 (22.7%)	13 (46.4%)	0.014	18 (31.6%)	17 (24.6%)	0.46

Signs	No. (%)			*p*	No. (%)		*p*
Breath rate > 24/min	10 (8%)	0	10 (35.7%)	0.00	4 (7.0%)	6 (8.7%)	0.83

Laboratory characteristics	Median (range)			*p*	Median (range)		*p*
White blood cell, × 10^9^/L	5.16 (3.91–6.30)	5.1 (3.0–6.1)	5.5 (4.6–6.9)	0.20	5.0 (3.6–6.4)	5.2 (4.4–6.2)	0.87
Neutrophil count, × 10^9^/L	3.20 (2.25–4.00)	3.1 (2.1–3.7)	3.8 (2.7.7)	0.002	3.1 (2.0–3.8)	3.3 (2.6–4.0)	0.98
Lymphocyte count, × 10^9^/L	1.20 (0.80–1.70)	1.42 (1.0–1.8)	0.8 (0.5–1.1)	< 0.001	1.2 (0.8–6.4)	1.2 (0.7–1.6)	0.39
Neutrophil–lymphocyte ratio	2.36 (1.70–4.17)	2.1 (1.6–2.9)	4.9 (2.1–7.8)	< 0.001	2.1 (1.7–4.0)	2.5 (1.8–4.2)	0.99
C-Reactive Protein, mg/dL	5.40 (1.30–24.0)	3.6 (1.0–9.7)	25 (5.4–66)	< 0.001	4.6 (1.0–21.9)	5.4 (2.0–25.0)	0.44
Procalcitonin, ng/mL	0.05 (0.00–0.08)	0.04 (0.00–0.05)	0.06 (0.03–0.11)	< 0.001	0.05 (0–0.05)	0.05 (0–0.09)	0.75
Lactic dehydrogenase, U/L	320.50 (200–456)	267 (177.5–412.0)	400 (321–590)	< 0.001	312 (200–428)	348 (200–460)	0.56
Creatine kinase, U/L	63.5 (40.0–107.8)	56.0 (38.0–89.5)	109.5 (76–125.9)	< 0.001	52 (39.5–120)	93.5 (54–213)	0.09
ALT, U/L	29.0 (18.0–45.8)	27.0 (17.0–40.5)	42.0 (24.2–59.3)	0.02	32 (21–45)	27.2 (17.0–46.0)	0.62
AST, U/L	25.9 (20.0–35.0)	24.0 (20.0–30.5)	29.1 (24.4–55.5)	0.03	26 (20–35)	24 (20–39)	0.37
Urea nitrogen, mmol/L	3.7 (3–4.9)	3.6 (3.0–4.7)	4 (2.8–5.3)	0.85	3.5 (3.0–4.4)	4 (3–5)	0.15
Creatinine, mmol/L	57.0 (48.0–66.8)	56.0 (45.5–66.0)	59.5 (49.7–80.5)	0.60	60 (48–67)	55 (47–66)	0.21
0xygenation index, mmHg	371.4 (324.1–377.9)	371.4 (371.0–381.0)	274.6 (225.0–362)	< 0.001	371.4 (342.9–371.4)	371.4 (323.8–381.0)	0.88

Imaging characteristics	No. (%)			*p*	No. (%)		*p*
Normal	17 (13.6%)	17 (17.5%)	0	0.01	7 (12.3%)	4 (5.8%)	0.2
Unilateral GGO	24 (19.2%)	23 (23.7%)	1 (3.6%)	< 0.001	13 (22.8%)	12 (2.9%)	0.45
Bilateral GGO	87 (69.6%)	62 (63.9%)	25 (89.3%)	< 0.001	36 (63.2%)	50 (72.5%)	0.27
Diffuse lesions in both lungs	3 (2.4%)	0	3 (10.7%)	0.02	1 (1.8%)	3 (4.3%)	0.15

Data are median (IQR) or No (%). GGO, ground glass opacities

### Clinical and laboratory features associated with disease progression or postponed viral shedding time

A total of 44 laboratory and clinical records on admission and during hospitalization were obtained and analyzed, including but not limited to the demographics, symptoms, signs, images, blood routine, immunocytochemistry, enzymatic and liver/renal function. These data were acquired within 24 h on admission. For the postponed viral shedding time discriminant model, six features (including one clinical feature, one demographic feature, one laboratory index and three therapeutic regimens) were selected to be the significant indicators of the postponed viral shedding time. They were listed as follows: the interval from the onset of symptoms to antiviral treatment (days) (x_2_), age (x_4_), CK (x_29_), Combined treatment of nebulized IFN-α with lopinavir/ritonavir (x_34_), Treatment with antibiotics (x_38_), Treatment with Methylprednisolone (x_41_) (Table 2).

**Table 2:** Treatment approaches among patients with different virus shedding of COVID-19 infection

	Virus shedding < 14 days (N = 56)	Virus shedding ≥ 14 days (N = 69)	*p*
	No. (%)		
Disease severity			
Severe/critical N (%)	8 (13.6%)	20 (29.0%)	0.24
Treatment approaches			
Treatment with Lobinavi/ritonavir alone	6 (10.5%)	8 (11.6%)	0.86
Treatment with Arbidol alone	7 (12.2%)	6 (8.7%)	0.53
Combined treatment of nebulized IFN-α with lopinavir–ritonavir	18 (31.6%)	9 (13.0%)	0.03
Combined treatment of nebulized IFN-α, with Arbidol	1 (1.8%)	5 (7.2%)	0.16
Combined treatment of nebulized IFN-α, lopinavir–ritonavir and Arbidol	12 (21.1%)	29 (42.0%)	0.04
Treatment with Oseltamivir phosphate alone	3 (5.3%)	3 (4.3%)	0.81
Treatment with moxifloxacin	12 (21.1%)	29 (42.0%)	0.04
Treatment with ribavirin	5 (8.8%)	6 (8.7%)	0.99
Treatment with Chinese traditional medicine (Xuebijing)	15 (26.3%)	29 (42.0%)	0.14
Treatment with Methylprednisolone	3 (5.3%)	15 (21.7%)	0.01
Treatment with γ-globulin	0	3 (4.3%)	0.12

Antiviral treatment course	Median (range)		*p*
	9 (4–12)	15 (7–19)	0.14

Data are median No (%)

*AUC* area under the curve

For the disease progression discriminant model, eleven features (including one demographic, two epidemiological features and one imaging, seven laboratory indexes) were significantly associated with the progression of severe disease. These features include the interval from Jan 1st, 2020 (the day for the first case emerged in Liaoning) to individualized onset of symptoms (x_2_), age (x_4_),imaging characteristics (x_5_), epidemiological history of short-term exposure to Wuhan (x_8_), and immune features [Lymphocyte counts (x_19_), leukocyte counts (x_20_), N/L ratio (x_21_), CRP (x_22_), PCT (x_23_)], Lactic dehydrogenase (x_24_),creatine kinase (x_29_).

No multi-collinearity was found in the screened variables, since VIF (Variance Inflation Factor) values of the screened dependent variables were all less than 10. Thus, the model passed the multi-collinearity test (Additional file 1: Tables S2, S3).

### Establishment and verification of the optimal discriminant models for disease progression and postponed viral shedding time

All samples were stratified and randomly divided into training and testing datasets, and all models were developed with fourfold cross-validation [10]. During construction of the discriminant model of postponed, the training datasets contained 55 postponed virus clearance cases and 45 non-postponed cases, while the testing dataset was consisted of 14 postponed virus clearance cases and 12 postponed cases. During construction of the discriminant model of disease progression, the training datasets contained 31 severe/critical cases and 69 non-severe cases, while the testing dataset was consisted of 8 severe/critical cases and 18 non-severe cases. Then, the discriminant models were constructed in training set via the selected indicators using several methods, including logistic regression, linear discriminant analysis, decision tree, K-nearest neighbor, and support vector machine method. The outcome was predicted in testing data set.

By analyzing total accuracy in both training and testing data set, the optimal discriminant models for the prediction of disease progression and postponed viral shedding time of COVID-19 infection were established via the logistic regression with the selected eleven and six features used as independent variables. In order to eliminate the influence of model overfitting on the prediction results and to improve the generalization ability of the model, L_2_ regularization $\left[ {\mathop {\min }\limits_{\beta ,C} \frac{1}{2}\beta^{T} \beta + C\sum\nolimits_{i = 1}^{n} {\log (\exp ( - y_{i} (X_{i} \beta + c) + 1))} } \right]$ was used to constrain the objective function in the logistic regression optimization process. Regarding the term of L_2_ regularization, *β* represents the coefficient in front of a variable. *C* represents the regularization parameter in cost function, and it controls the strength of L_2_ regularization. *X*_*i*_ represents the value of the attribute variable and *c* represents the constant term.

"Viral shedding postponed or not" was used as a dependent variable (y_1_) for logistic regression, which yielded the equation:

$$Ln\left(\frac{p}{1-p}\right)=-0.244+0.2829{x}_{1}+0.2306{x}_{4}+0.234{x}_{28}-0.2847{x}_{34}+0.3084{x}_{38}+0.3025{x}_{41}.$$

Within the formula, p represents the probability of the patient being judged positive (viral shedding postponed).

"Severe or not" was used as a dependent variable (y_2_) for logistic regression. This yielded the equation:

$$Ln\left(\frac{p}{1-p}\right)=-0.348-0.099{x}_{2}+0.0945{x}_{4}+0.1176{x}_{5}+0.0398{x}_{8}-0.1646{x}_{19}+0.0914{x}_{20}+0.1254{x}_{21}+0.1397{x}_{22}+0.0814{x}_{23}+0.1294{x}_{24}+0.1099{x}_{29}.$$

Within the formula, p represents the probability of the patient being judged positive (severe disease). Notably, all data needs to be equally standardized [$x^{*} = \frac{x - \mu }{\sigma }$] before being plugged into the equation, and the corresponding mean and standard deviation were determined and shown in Additional file 1: Tables S4, S5.

### Performance of the discriminant models

**Fig. 1:**
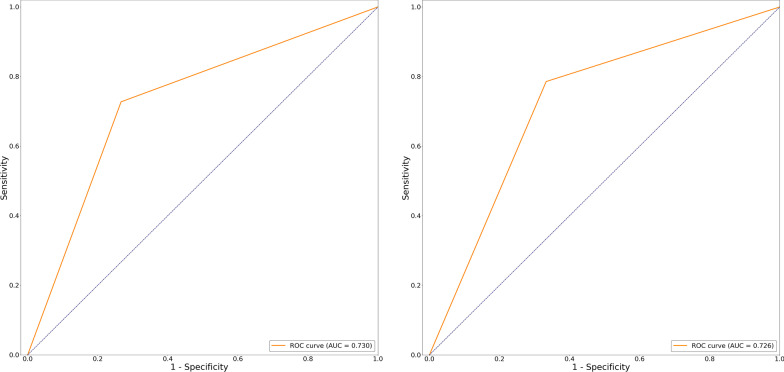
AUROC curve of the postponed viral shedding discriminant model for training set (**a**) and testing set (**b**). *AUROC* area under receiving operating curve

When applying the discriminant model of postponed viral shedding time onto the validation of the training set (Tables 3, 4; Additional file 1: Table S6), sensitivity was 0.727, specificity was 0.733, positive predictive value was 0.769, and negative predictive value was 0.688. In the test set, sensitivity was 0.786, specificity was 0.667, with positive predictive value 0.733 and negative predictive value 0.727. Recall rate was 78.6%. Accuracy was 0.732. The AUROC of the combinations of 7 clinical features was 0.73 in the training dataset (Fig. 1a) and 0.73 in the testing dataset (Fig. 1b).

**Table 3:** The summary of primary discriminant screening models for virus shedding of Covid-19 infection

	Discriminant models for postponed viral shedding time									
	Training set with cross-validation					Test set with cross-validation				
	Logistic regression	Linear discriminant analysis	K-nearest neighbor	Support vector machine	Decision tree	Logistic regression	Linear discriminant analysis	K-nearest neighbor	Support vector machine	Decision tree
AUC	73.0	59.1	35.7	45.0	40.9	73.0	57.9	40.6	44.6	39.0
Sensitivity, %	72.7	62.2	45.9	53.9	39.5	78.6	59.3	50.4	30.9	42.0
Specificity, %	73.3	36.4	39.9	40.7	35.5	66.7	39.4	49.9	49.7	25.0
Positive predictive value, %	76.9	60.1	48.9	50.3	25.2	73.3	50.4	45.2	41.5	32.9
Negative predictive value, %	68.8	43.4	30.4	39.9	30.4	72.7	40.0	49.8	55.4	29.3
Accuracy	–	–	–	–	–	73.2	50.3	60.3	30.4	20.3
Recall rate	–	–	–	–	–	78.6	51.3	45.0	34.9	29.9

*AUC* area under the curve

**Table 4:** The summary of primary discriminant screening models for different severities of Covid-19 infection

	Discriminant models for disease progression									
	Training set with cross-validation					Test set with cross-validation				
	Logistic regression	Linear discriminant analysis	K-nearest neighbor	Support vector machine	Decision tree	Logistic regression	Linear discriminant analysis	K-nearest neighbor	Support vector machine	Decision tree
AUC	82.9	60.9	47.6	26.3	43.4	81.9	62.3	46.0	39.0	36.9
Sensitivity, %	77.4	52.0	40.3	36.5	49.1	75	50.4	49.8	40.1	41.9
Specificity, %	88.4	69.9	34.4	24.4	30.4	88.9	60.2	35.1	35.2	29.8
Positive predictive value, %	75	46.7	45.6	45.3	51.2	75	59.1	55.5	33.0	30.5
Negative predictive value, %	89.7	55.9	40.3	30.4	37.4	88.9	58.3	40.1	50.3	40.3
Accuracy	–	–	–	–	–	84.6	55.4	35.4	41.3	34.5
Recall rate	–	–	–	–	–	75	60.0	40.1	29.9	35.3

**Fig. 2:**
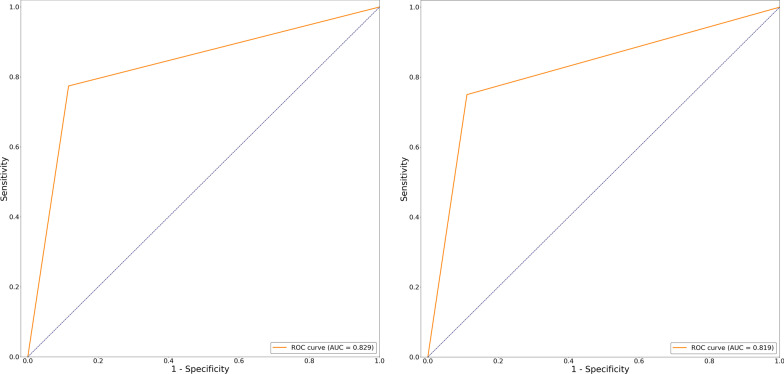
AUROC curve of the disease progression discriminant model for training set (**a**) and testing set (**b**). *AUROC* area under receiving operating curve

According to the confusion matrix of discriminant model for disease progression (Tables 3, 4; Additional file 1: Table S7), during the validation of the training set, sensitivity was 0.774, specificity was 0.884, positive predictive value was 0.75, and negative predictive value was 0.897. In the test set, sensitivity was 0.75, specificity was 0.889, with positive predictive value 0.75and negative predictive value 0.889. The recall rate was 75%, and accuracy was 0.846. The AUROC was also constructed to evaluate the effectiveness of the discriminant models The AUROC of the combinations of 11 demographic, clinical and imaging/laboratory features was 0.829 (Fig. 2a) in the training dataset and 0.819 (Fig. 2b) in the testing dataset.

## Discussion

This retrospective study tentatively developed two discriminant models consisting of several clinical and epidemiological features that could be quickly obtained on admission. To date, a certain number of models predict the risk of severe COVID-19 has been developed [5, 11]. However, there is geographic discrepancy of severity and mortality rate in patients with COVID-19 infection, and most of the previous prediction models were established to predict survival risk or progression to severe or critical state in the south part of China [5, 11]. Few of them were designed to predict the postponed viral shedding time. Besides, since there is no consensus about the therapeutic regimen of COVID-19 [12], the models established previously mainly consisted of variables that extracted from the clinical and laboratory parameters, with few of them incorporated epidemiological features or therapeutic regimen [11]. To the best of our knowledge, for the first time, the impact of epidemiological features and therapeutic regimen on the disease progression and postponed viral shedding time were described and integrated into the predicted polynomial equations among patients confirmed as COVID-19 infection.

Overall, these two discriminant models in the present study was demonstrated to have satisfactory sensitivity (> 72.00%) and specificity (> 73.00%), and they can be used as early warning tools to robustly and effectively predict the postponed viral shedding time and the severe/critical progression in patients with COVID-19 infection upon admission. A medical staff can easily predict in advance using these two discriminant models and conduct a timely and optimal medical intervention at an early stage.

SARS-CoV-2 in the respiratory tract, especially sputum, has been observed to be associated with a prolonged viral shedding and high viral load, when compared with the stool specimens [13]. In this study, the median viral shedding time was 14 days (IQR, 11–19). In order to effectively control the transmission resources on the imported cities or regions, it is essential to identify factors that associated with the COVID-19 PCR negative conversion time and to establish a prediction model that could individually estimate the risk of postponed viral shedding time among patients with COVID-19 upon admission to hospitals or shelters. In this study, we initially selected 43 variables probably associated with disease progression and postponed viral shedding time respectively according to the published literatures [4–7], then detected the risk factors by F-test and *χ*^2^ test analysis. As a result, 6 variables were identified as discriminatory factors and were devised to discriminant models for prediction of postponed viral shedding time of COVID-19 infection. Both older age and delayed antiviral treatment could give rise to the postponed viral shedding time. Consistently, these results were in accordance with another previous study indicated that the time from symptom onset to viral clearance slightly increased with age [14]. Besides, the association between delayed initiation of antiviral treatment and the prolonged virus shedding for influenza A (H7N9) and SARS-CoV-2 was also observed in previous studies, indicating that timely initiation of antiviral treatments necessary for viral clearance [15–17]. In addition, dual-antiviral therapy of nebulized IFN-α with lopinavir/ritonavir (x_33_) was negatively associated with the viral shedding time, whereas treatment with antibiotics (x_37_) and methylprednisolone (x_40_) were related to postponed viral shedding time. This was consistent with previous studies which demonstrated that SARS-CoV-2 was more susceptible to IFNs when compared to SARS-CoV, as the inhalation of Interferon-α (IFN-α) 2b could reduce the infection rate significantly and it can be used for prophylaxis of SARS-CoV-2 infection [18–20]. In addition, although Lopinavir/ritonavir (Kaletra) presented controversial therapeutic effects as compared to the standard care in vivo [22–25], it was found to have anti-SARS-CoV efficacy in vitro [21], thus Lopinavir/ritonavir has been recommended by the National Health Commission of China for the treatment of COVID-19 in the early period of 2020 [26]. To the best of our knowledge, for the first time this study observed that combined treatment of IFN-α inhalation and lopinavir/ritonavir was related to the shortened viral shedding time of COVID-19. Nevertheless, there is no additional benefit on virus clearance from an extra use of Arbidol, when combined with IFN-α and lopinavir/ritonavir. Anyway, statistical analysis can only stress association but cannot explain causality. A recent study also observed that early initiation of dual-antiviral treatment with lopinavir/ritonavir + IFN-α combination therapy could help shorten the duration of SARS-CoV-2 shedding when compared with triple antiviral treatment (opinavir/ritonavir + IFN-α + arbidol) [17]. This conclusion may provide a rationale for clinicians to optimize and to early initiate the antiviral treatments. In a previous study, the administration of corticosteroids has been observed to bring benefits for patients infected by COVID-19, since will prevent the use of mechanical ventilation and reduce the mortality of them [27]. Conversely, in this study, treatment with antibiotics (x_37_) and methylprednisolone (x_40_) could give rise to the postponed viral shedding time. Some previous studies have also observed that high-dose of corticosteroids was associated with increased mortality and longer viral shedding in patients with influenza A (H7N9) viral pneumonia and MERS [28–30]. Systemic corticosteroids could increase the risk of opportunistic infections (such as bacterial or fungal) that occur secondary to immunosuppression, and eventually hinders the virus clearance ability [31]. Besides, potential bacterial infections secondary to influenza viral infection that has been commonly seen in this study (32.8%) could also prolong the viral shedding time, as indicated by the evidence that the use of antibiotics was associated with postponed viral shedding time.

Noteworthy, this study for the first time observed that intervals from the first case emerged in Liaoning province (Jan 22st, 2020) to the individualized onset of symptoms (x_2_) could serve as an important prognostic feature in our early-warning model of disease progression. Indeed, nearly half of the confirmed cases at the early stage (in January) of COVID-19 outbreak were severe cases, whereas in the latter period (after February), the percentage of non-severe cases became dominant (76.9%) in Liaoning province. One explanation is that pathogens tend to reduce their virulence overtime in order to maximize their between-host transmission, which could result in the gradually lowered severity of COVID-19 infection on the imported regions [32, 33]. Besides, the human intervention efforts in China, such as to promptly admit suspected patients to the designated shelter hospitals, have effectively contributed to the decreased number of severe cases of COVID-19. Interestingly, short-term exposure to the epidemic area (Wuhan) during traveling or transfer could also result in a higher likelihood of progressing into severe stage of disease. Those transiently migrant individuals might have been primed by one or more prior coronavirus exposures during traveling or transfer around the epidemic area, thus have experienced the effects of antibody dependent enhancement (ADE) antigenic epitope heterogeneity due to antigenic epitope heterogeneity [34]. ADE hinders the ability to manage inflammation and result in disease progression. Another explanation would be attributed to the SARS-CoV-2 strains of L type, which are evolutionarily more aggressive and contagious. This virus strain of L type with altered virulence could probability be the underlying causal pathogen for patients who acquired infections via short-term exposure to the epidemic area [35].

In our discriminant models, both immune features (lymphocytes, neutrophils, N/L ratio, CRP) and enzymatic index (LDH, CK) obtained on admission were observed as the most significant prognostic factors for disease severity. These are consistent with the well-established within-host model in previous literature, which describes the interactions between SARS-CoV-2, host pulmonary epithelial cells and cytotoxic T lymphocyte cells [36]. Our results illustrate an earlier exhibition of abnormal laboratory features prior to the disease progression [37, 38]. Consistent with previous studies, our study revealed that advanced age in a strong risk factor for more severe COVID-19 infection [39–41]. This suggests that more intensive surveillance is necessary in elderly patients.

Anyway, some limitations should be noted in this study. First, the study design was retrospective, and the sample size may be insufficient for characterization of an entire population. However, by including all patients from eight designated hospitals throughout Liaoning province, we considered patients recruited in this study are representative of cases diagnosed with COVID-19 in Liaoning, China. Secondly, not enough severe/critical cases were recruited for the present study. This was possibly because the fatality rate of patients infected by COVID-19 in Liaoning province was lower (1.6%) than the whole national average level (3.2%) [42], and not resembling the previous studies from Wuhan [2, 43]. Thirdly, we only included the initial antiviral treatments as factors for prolonged shedding duration, so as to minimize the bias of different efficacy caused by different treatment courses as much as possible. This discrepancy may have had an unknown influence on the efficacy of the models.

## Conclusion

The discriminant models reported here is the first attempt of its kind to develop an early warning tool for both postponed viral shedding time and disease progression in the northeast area of China. We believe that these models can help to judge the disease progression early enough in a great number of patients with COVID-19 infection, and this early judgment can facilitate a timely medical intervention, which will ultimately reduce the mortality of COVID-19.

## Supplementary Information

**Additional file 1:** The variable assignment and multi-collinearity analysis of independent variables in two models.

## Data Availability

The datasets used and/or analyzed during the current study are available from the corresponding author on reasonable request.

## Abbreviation

COVID-19: Coronavirus disease 2019
