# Supplementary material for: Discriminant models for the prediction of postponed viral shedding time and disease progression in COVID-19
Source: BMC Infect Dis. 2022 Apr 11;22:366. doi: 10.1186/s12879-022-07338-x (PMC8996205; doi:10.1186/s12879-022-07338-x)
Supplement: Supplementary file 1 — Additional file 1: The variable assignment and multi-collinearity analysis of independent variables in two models. [file 12879_2022_7338_MOESM1_ESM.docx]

**Supplementary table 1. The variable assignment.**

| Factor | Variable | Quantification |
| --- | --- | --- |
| x_1_ | Interval from onset of symptoms to antiviral treatment | The days between disease onset and being treated |
| x_2_ | Interval from Jan 1^st^ ,2020 to onset of symptoms | The days between the first case emerged in Liaoning to individualized onset of symptoms |
| x_3_ | Gender | Female=0, Male=1 |
| x_4_ | Age | Year of birth to 2019 |
| x_5_ | Imaging characteristics | None = 0, Unilateral GGO = 1, Bilateral GGO = 2, Diffuse lesions in both lungs = 3 |
| x_6_ | Epidemic feature with long-term exposure to Wuhan | No = 0, Yes =1 |
| x_7_ | Epidemic feature with local- transmitted history | No = 0, Yes =1 |
| x_8_ | Epidemic feature with short-term exposure to Wuhan | No = 0, Yes =1 |
| x_9_ | Respiratory symptoms | No = 0, Yes =1 |
| x_10_ | Digestive symptoms | No = 0, Yes =1 |
| x_11_ | Symptoms with general malaise | No = 0, Yes =1 |
| x_12_ | Fever | No = 0, Yes =1 |
| x_13_ | Comorbidities with chronic respiratory disease | No = 0, Yes =1 |
| x_14_ | Comorbidities with hypertension | No = 0, Yes=1 |
| x_15_ | Comorbidities with diabetes mellitus | No = 0, Yes=1 |
| x_16_ | Surgery history | No = 0, Yes=1 |
| x_17_ | Other comorbidities | No = 0, Yes=1 |
| x_18_ | WBC | Actual value |
| x_19_ | lymphocyte counts | Actual value |
| x_20_ | leucocyte counts | Actual value |
| x_21_ | leucocyte/lymphocyte ratio | Actual value |
| x_22_ | CRP | Actual value |
| x_23_ | PCT | Actual value |
| x_24_ | LDH | Actual value |
| x_25_ | AST | Actual value |
| x_26_ | ALT | Actual value |
| X_27_ | Cr | Actual value |
| x_28_ | Urea | Actual value |
| x_29_ | CK | Actual value |
| x_30_ | oxygenation index | Actual value |
| x_31_ | Severity of COVID-19 infection | Non-severe = 0, Severe =1 |
| x_32_ | Treatment with Lobinavi/ritonavir alone | No = 0, Yes=1 |
| x_33_ | Treatment with Arbidol alone | No = 0, Yes=1 |
| x_34_ | Combined treatment of nebulized IFN-α with lopinavir–ritonavir | No = 0, Yes=1 |
| x_35_ | Combined treatment of nebulized IFN-α, with Arbidol | No = 0, Yes=1 |
| x_36_ | Combined treatment of nebulized IFN-α, lopinavir–ritonavir and Arbidol | No = 0, Yes=1 |
| x_37_ | Treatment with Oseltamivir phosphate alone | No = 0, Yes=1 |
| x_38_ | Treatment with moxifloxacin | No = 0, Yes=1 |
| x_39_ | Treatment with ribavirin | No = 0, Yes=1 |
| x_40_ | Treatment with Chinese traditional medicine | No = 0, Yes=1 |
| x_41_ | Treatment with Methylprednisolone | No = 0, Yes=1 |
| x_42_ | Treatment with γ-globulin | No = 0, Yes=1 |
| x_43_ | Antiviral treatment course | The days for antiviral treatment |
| y_1_ | Virus shedding time | Virus conversion time less than 14 days=0,  Virus conversion time equal to or more than 14 days =1 |
| y_2_ | Severity of disease | Non-severe=0, Severe=1 |

GGO, ground glass opacities.

**Supplementary table 2. Multi-collinearity analysis of independent variables in discriminant model of disease progression**

| Variable | VIF value | Variable | VIF value | Variable | VIF value | Variable | | VIF value |
| --- | --- | --- | --- | --- | --- | --- | --- | --- |
| x_2_ | 1.34 | x_8_ | 1.19 | x_21_ | 7.05 | x_24_ | 1.60 | |
| x_4_ | 1.47 | x_19_ | 3.93 | x_22_ | 1.60 | x_29_ | 1.13 | |
| x_5_ | 1.07 | x_20_ | 5.58 | x_23_ | 1.07 |  |  | |

Vif value, variance inflation factor value;

**Supplementary table 3. Multi-collinearity analysis of independent variables in discriminant model of postponed virus shedding time**

| Variable | VIF value | Variable | VIF value |
| --- | --- | --- | --- |
| x_1_ | 1.09 | x_36_ | 1.21 |
| x_4_ | 1.11 | x_38_ | 1.18 |
| x_28_ | 1.04 | x_41_ | 1.25 |
| x_34_ | 1.18 |  |  |

**Supplementary table 4. Corresponding mean and standard deviation of postponed virus shedding model variables**

| Variables | Mean(μ) | Standard deviation(σ) |
| --- | --- | --- |
| x_1_ | 4.80 | 3.93 |
| x_4_ | 44.65 | 17.29 |
| x_28_ | 4.45 | 4.00 |
| x_34_ | - | - |
| x_38_ | - | - |
| x_41_ | - | - |

**Supplementary table 5. Corresponding mean and standard deviation of disease progression model variables**

| Variables | Mean(μ) | Standard deviation(σ) |
| --- | --- | --- |
| x_2_ | 12.97 | 8.51 |
| x_4_ | 44.65 | 17.29 |
| x_5_ | - | - |
| x_8_ | - | - |
| x_19_ | 1.31 | 0.73 |
| x_20_ | 3.49 | 1.87 |
| x_21_ | 3.75 | 4.83 |
| x_22_ | 19.21 | 28.86 |
| x_23_ | 0.06 | 0.08 |
| x_24_ | 354.82 | 200.22 |
| x_29_ | 110.85 | 148.53 |

**Supplementary table 6. The confusion matrix of logistic regression discriminant model for postponed virus shedding**

| Training | Predicted Negative | Predicted Positive |
| --- | --- | --- |
| Real Negative | 33 | 12 |
| Real Positive | 15 | 40 |
| Testing | Predicted Negative | Predicted Positive |
| Real Negative | 8 | 4 |
| Real Positive | 3 | 11 |

**Supplementary table 7. The confusion matrix of logistic regression discriminant model for disease progression (Training set with cross-validation)**

| Training | Predicted Negative | Predicted Positive |
| --- | --- | --- |
| Real Negative | 61 | 8 |
| Real Positive | 7 | 24 |
| Testing | Predicted Negative | Predicted Positive |
| Real Negative | 16 | 2 |
| Real Positive | 2 | 6 |
